# Transdiagnostic Factors and Their Relationship to Post-Traumatic Stress and Psychopathology in Clinical Populations

**DOI:** 10.62641/aep.v54i1.2059

**Published:** 2026-02-15

**Authors:** Olga Ribera-Asensi, Aiara Rodríguez-Fernández, Marián Pérez-Marín, Selene Valero-Moreno

**Affiliations:** ^1^Departamento de Psiquiatría, Hospital Arnau de Vilanova, 46015 Valencia, Spain; ^2^Faculty of Psychology and Speech Therapy, University of Valencia, 46010 Valencia, Spain; ^3^Personality, Assessment and Psychological Treatments Department, Faculty of Psychology and Speech Therapy, University of Valencia, 46010 Valencia, Spain

**Keywords:** psychopathology, traumatic stress disorder, emotion regulation, resilience, social support, personality

## Abstract

**Background:**

High comorbidity among mental disorders challenges the utility of categorical classifications. The transdiagnostic perspective focuses on common psychological processes, potentially overcoming these limitations. The aim of this study was to explore transdiagnostic factors related to post-traumatic stress and psychopathology in a clinical sample.

**Methods:**

Sixty-one patients (73.8% women; aged 20–66 years, M = 43.79, standard deviation (SD) = 12.75) from the Adult Mental Health Unit of the Hospital Clínico Universitario of Valencia were assessed on symptomatology (post-traumatic stress and psychopathology) and psychological variables (emotional dysregulation, resilience, personality, social support, and stressful life events).

**Results:**

Our results indicated elevated post-traumatic stress scores (M = 70.49, SD = 20.33), suggesting clinically significant distress, together with low exposure to stressful life events (Median (Mdn) = 2.00; interquartile range (IQR) = 2.00). Post-traumatic stress and psychopathology were positively correlated with emotional dysregulation and negatively with resilience, social support, extraversion, and conscientiousness. Higher post-traumatic stress and psychopathology were linked to low social support, low extraversion, and high emotional dysregulation. Emotional dysregulation emerged as a key moderating variable, potentially diminishing the protective effects of resilience on post-traumatic stress.

**Conclusions:**

Overall, findings support the transdiagnostic approach, highlighting that factors like emotion regulation contribute not only to symptom development but also critically influence how risk and protective factors affect mental health outcomes, emphasizing the importance of targeting these processes in clinical intervention and prevention efforts.

## Introduction

According to the World Health Organization (WHO), in 2019, 970 million people worldwide suffered from a mental disorder [1]. Mental disorders are often defined categorically, based on the presence or absence of symptoms according to quantitative criteria set out in diagnostic manuals such as the DSM-5-TR [2] or ICD-11 [3]. In Spain, the most common disorders in primary care are anxiety disorders (10.65% of the population), sleep disorders (8.16%), and depressive disorders (4.78%) [4], with comorbidity rates between anxiety and depression exceeding 65% [5]. On the other hand, conditions such as post-traumatic stress disorder (PTSD) have a prevalence of 3.50% in the general population [6], and comorbidity rates ranging from 50% to 80% with anxiety, depression, alcohol abuse, or somatization [7, 8].

Categorical diagnoses present several practical limitations. One major issue is the high rate of comorbidity, which is associated with worse treatment outcomes, reduced patient quality of life, and higher levels of functional impairment [7, 9, 10]. To overcome these limitations, dimensional approaches such as the transdiagnostic model focus on shared causal or maintaining processes underlying mental disorders [11]. Similarly, other authors have supported this perspective through dimensional personality models [12] and transdiagnostic treatment protocols like the Unified Protocol for Emotional Disorders [13].

Currently, emotional disorders have the strongest evidence within transdiagnostic models, which emphasize common vulnerability factors underlying various forms of psychopathology. In this context, conditions such as PTSD have shown associations with transdiagnostic vulnerability variables linked to emotional disorders. Among these variables, personality traits play an important role. Certain traits have been consistently associated with mental health outcomes [13]. For example, extraversion is negatively related to depression and social dysfunction and positively linked to mania [14]. Lower levels of extraversion or conscientiousness have also been associated with post-traumatic symptoms [15], suggesting that specific personality configurations may predispose individuals to greater vulnerability following trauma exposure.

Another central transdiagnostic factor is emotion regulation, defined as the adaptive management of emotional responses [16]. Dysfunctional emotion regulation has been associated with greater PTSD symptom severity [17], suggesting that difficulties in emotion regulation act both as a risk factor and a consequence of PTSD symptoms, increasing the risk of symptom persistence [18].

In addition to emotion regulation, several protective and risk factors have been identified in the transdiagnostic literature. Resilience, defined as the capacity to recover and achieve positive outcomes after adverse events [19], serves as a protective factor for both emotional disorders [20, 21] and PTSD [22]. Resilience has also been shown to moderate the impact of stress [23]. Similarly, social support has demonstrated a protective effect against the development and maintenance of emotional disorders [24] and PTSD [25].

Experiencing stressful life events has consistently been identified as a robust predictor of depressive and anxiety symptoms and, most notably, of PTSD symptomatology [26]. However, not all individuals exposed to stress develop psychopathology, which highlights the role of moderating factors that influence this relationship. Emotion regulation and resilience have been identified as key transdiagnostic constructs involved in stress adaptation and mental health [27, 28].

Emotion regulation refers to the processes by which individuals influence the experience and expression of emotions, and its adaptive or maladaptive use can shape responses to stress [27]. Specifically, maladaptive strategies such as emotional suppression tend to exacerbate stress responses and psychopathological symptoms [28]. Conversely, adaptive regulation strategies may buffer the psychological impact of stress exposure. Resilience, in turn, reflects the capacity to recover or maintain psychological stability in the face of adversity. Theoretical models of resilience conceptualize it as a protective mechanism that mitigates the effects of emotion dysregulation and stress on mental health outcomes [29].

Therefore, in the present study, emotion regulation was examined as a moderator of the relationship between stressful life events and psychopathology, whereas resilience was conceptualized as a protective moderator that buffers the impact of emotion dysregulation on psychopathology and post-traumatic stress. This dual framework is grounded in contemporary transdiagnostic models of emotional functioning, which emphasize the dynamic interplay between regulation and resilience as core mechanisms underlying vulnerability and adaptation across mental disorders [30, 31]. In view of the above, this study aims to analyze how transdiagnostic factors—including emotion regulation, personality traits, resilience, stressful life events, and social support—are associated with post-traumatic stress and psychopathology in a clinical adult population in Spain, using correlational, qualitative QCA, and PROCESS models. Based on this objective, the following hypotheses were proposed: H1 Symptoms of post-traumatic stress and psychopathology will be associated with stressful life events, emotion dysregulation, low resilience, low extraversion, low conscientiousness, and low social support, as evidenced by correlation and QCA analyses; H2. The impact of stressful life events on post-traumatic stress and psychopathology will be moderated by emotion regulation; H3. The effect of emotion regulation on post-traumatic stress and psychopathology will be moderated by resilience.

## Materials and Methods

### Participants

The study included 61 adult patients who were consecutively referred by their clinicians to the Adult Mental Health or Primary Care services of the Hospital Clínico Universitario de Valencia between 2021 and 2023. Eligible participants were adults presenting with clinically significant emotional distress requiring psychological care and meeting DSM-5 criteria for anxiety, depressive, obsessive-compulsive, trauma-related, or somatic disorders. Patients were excluded if they had cognitive impairments, severe medical illnesses, or diagnoses of substance-induced disorders, bipolar disorder, schizophrenia, addictions, neurodevelopmental, neurocognitive, personality, or eating disorders.

### Instruments

An ad-hoc questionnaire to assess demographic (gender, age, marital status, educational level, employment history and status, and family socio-economic status) and clinical variables (past and current psychological, psychiatric, and relevant medical treatments) was used.

Post-traumatic stress: was assessed using the Spanish version [32] of the PTSD Checklist – Civilian Version (PCL-5) [33]. The questionnaire consists of 20 items that assess symptoms present following direct or indirect exposure to a traumatic event, using a 5-point Likert scale in which 0 corresponds to “strongly disagree” and 4 to “strongly agree”. A total post-traumatic stress score is obtained by summing the scores of each item, such that the higher the score, the greater the level of stress. The total score ranges from 0 to 80. Regarding psychometric properties, the adaptation study reported good factorial validity [34]. In the present study, internal consistency was also adequate (α = 0.94).

Psychopathology: psychopathological symptoms were assessed using the Spanish adaptation [35] of the Symptom Checklist-90-Revised (SCL-90-R) [36]. The questionnaire is composed of 90 items that evaluate psychopathology on a 5-point Likert scale, where 0 corresponds to “not at all” and 4 to “extremely”. The Global Severity Index (GSI) is obtained by calculating the mean score of all items, resulting in a total score ranging from 0 to 4. Regarding its psychometric properties, González de Rivera *et al*. [35] reported satisfactory reliability (α ranging from 0.69 to 0.85). In the present study, internal consistency was also good, with α values ranging from 0.73 to 0.92. In this study, only the Global Severity Index was used.

Extraversion and Conscientiousness were measured using the Spanish version of the NEO Five-Factor Inventory (NEO-FFI) [37]. The inventory consists of 60 items, of which 12 belong to the Extraversion dimension and 12 to the Conscientiousness dimension, answered on a 5-point Likert scale, where 0 corresponds to “strongly disagree” and 4 to “strongly agree”. Although the scale includes all five personality factors, in this study only the Extraversion and Conscientiousness dimensions were used, as these are the traits most commonly associated with stress symptomatology in the literature. Scores for each dimension are obtained by summing the corresponding item scores. Extraversion scores range from 0 to 48, and Conscientiousness scores range from 0 to 48. Regarding psychometric properties, the Spanish adaptation has shown very good reliability (α between 0.82 and 0.90). In the present study, the following internal consistency values were obtained: Extraversion α = 0.86; Conscientiousness α = 0.82.

Emotion dysregulation: was measured using the Spanish adaptation [38] of the Difficulties in Emotion Regulation Scale (DERS) [39]. The scale consists of 28 items and assesses difficulties in the adaptive management of emotions using a 5-point Likert scale, where 1 corresponds to “almost never” and 5 to “almost always”. It has a multidimensional structure composed of five dimensions: lack of emotional control, non-acceptance, inattention, confusion, and interference. Scores for each dimension are obtained by summing the corresponding item scores, and the total score is obtained by summing all dimension scores. The total score ranges from 28 to 140. Regarding psychometric properties, Hervás and Jódar [38] reported good reliability results. In this study, internal consistency was good for the total scale (α = 0.93) and subscales (α = 0.73–0.92).

Resilience: was assessed using the 10-item reduced Spanish version of the Connor-Davidson Resilience Scale (CD-RISC-10) [40]. This scale provides a total resilience score reflecting positive adaptation following adversity, ranging from 0 to 40. Internal consistency in this study was good (α = 0.85).

Social support: was assessed using the Spanish version [41] of the Duke Functional Social Support Questionnaire This 11-item measure evaluates perceived emotional and confidential support on a 5-point Likert scale. Total scores range from 11 to 55, with higher scores indicating greater perceived support. Internal consistency in this study was good (α = 0.85).

Stressful life events: was evaluated using the Spanish version [42] of the List of Threatening Experiences (LTE) [26]. The questionnaire consists of 12 dichotomous response items (yes/no) that assess the experience of significant and prevalent stressful events during the last 6 months. The total score is obtained by summing the item scores; therefore, higher scores indicate a greater presence of stressful life events. The total score ranges from 0 to 12. The psychometric properties of the original study show an internal consistency of 0.44. In the present study, internal consistency was good (α = 0.85).

### Procedure

This study employed a cross-sectional correlational design. Data collection was carried out between 2021 and 2023 in a mental health service at a hospital. Patients, referred to by their clinicians after their first visit, were assessed in person at adult mental health or primary care centers. All self-administered reports were conducted through individual interviews with each of the participating subjects and both inclusion and exclusion criteria were strictly applied.

**Fig. 1.**
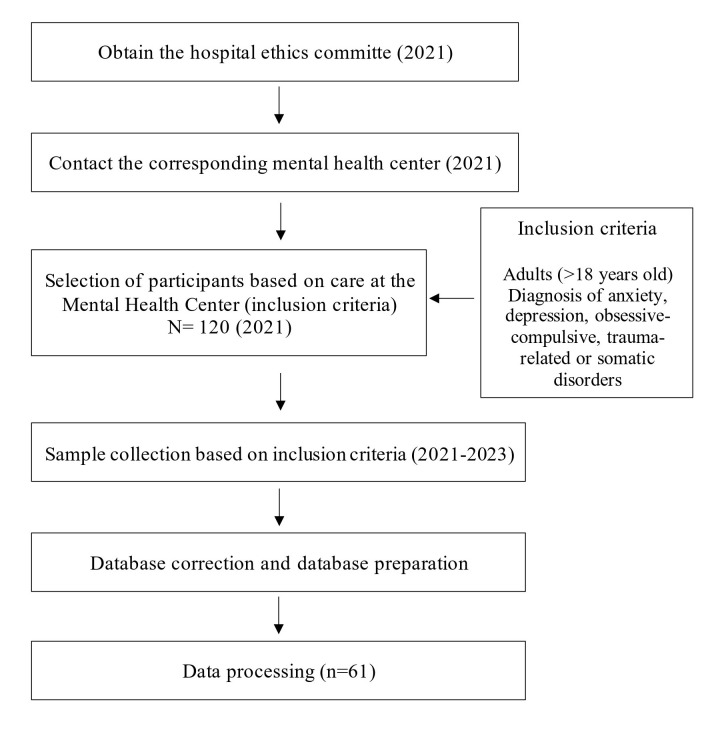
**Study procedure diagram**.

The study protocol was reviewed and approved by the Hospital Clínico Universitario Ethics Committee (Ceim2021-223). Written informed consent was obtained from the participants. All procedures performed in this study involving human participants were in accordance with the ethical standards of the institutional research committee and with the Declaration of Helsinki and its subsequent amendments (Fig. 1).

### Data Analysis

Data analyses in this study were conducted using various statistical software: IBM SPSS Statistics for Windows, version 28.0 (IBM Corp., Armonk, NY, USA); the fsQCA program, version 3.0 (Ragin, Charles C., Tucson, AZ, USA); and the PROCESS macro for SPSS, version 3.2 (Andrew F. Hayes, Columbus, OH, USA). A variety of statistical procedures were executed, depending upon the nature of the variables and hypotheses postulated. The reliability of the scales was assessed using Cronbach’s α coefficient, and descriptive statistics were calculated to obtain the mean and standard deviation of the variables. Prior to hypothesis testing, the assumptions of normality were evaluated for all continuous variables using the Kolmogorov–Smirnov and Shapiro-Wilk test. In addition, skewness and kurtosis indices, as well as medians and interquartile range, were calculated to further assess the distribution of the data. Since some variables followed a normal distribution while others did not, Spearman’s correlation coefficient was employed to examine the relationships between variables. Categorical variables were summarized using frequencies and percentages and analyzed with the chi-square test or Fisher’s exact test when required. Furthermore, the fuzzy data comparative qualitative-quantitative analysis (fsQCA) method was employed [43]. This advanced technique enables the exploration of how distinct combinations of causal conditions influence a given outcome, grounded in Boolean logic and the principle of equifinality. The latter posits that the achievement of a particular outcome can be realized through disparate combinations of conditions. In contrast to conventional linear models, which analyze the individual impact of each variable, fsQCA focuses on the interactions between conditions, thereby enabling the comprehension of how these conditions combine to generate a result.

In this analysis, two types of conditions are distinguished: necessary conditions, which must always be present for a result to occur, and sufficient conditions, which, although not always necessary, can produce the result if they are present in combination with other conditions. The transformation of data into fuzzy sets is achieved through the implementation of recalibration thresholds based on percentiles. The 10th, 50th, and 90th percentiles are frequently utilized to establish cut-off points [44]. The 10th percentile (0) indicates low agreement, the 50th (0.5) represents an intermediate level of agreement, and the 90th (1) reflects high agreement. This recalibration is imperative, particularly when dealing with continuous variables or surveys comprising multiple items (as in the present study, where all variables were continuous or multi-item), as it guarantees that observations are accurately allocated to fuzzy sets. The process entails the establishment of a truth table that enumerates all possible combinations of causal conditions and their empirical outcomes. The subsequent table illustrates the three types of solutions that are generated: complex, which is the most restrictive and detailed; parsimonious, which is the most generalized and least restrictive; and intermediate, which balances both and is the most commonly used in empirical studies [45, 46]. In order to assess the results, two key indicators must be taken into consideration: coverage, which is defined as the percentage of cases explained by a combination of conditions, and consistency, which is defined as the reliability of the model. The presence of high consistency (≥0.74) signifies that the model is reliable and that the combinations of conditions possess a high degree of predictive power for the observed outcome. It should be noted that the calibration thresholds used in fsQCA (90th, 50th, and 10th percentiles) follow established methodological guidelines, while in moderation analyses, the PROCESS macro automatically calculates the cut-off points (mean ± SD). Given that these procedures follow different methodological logics, these thresholds are not expected to coincide. For the purposes of interpreting and discussing the results, we have standardized on the thresholds derived from the PROCESS moderation analyses (M ± SD) as the core reference. FsQCA thresholds are used solely for calibration purposes. This approach ensures a consistent framework for interpreting high, medium, and low levels across variables while maintaining the methodological rigor of both analytical techniques. The Discussion section explicitly considers both methods while taking these threshold differences into account.

Descriptive analyses of the variables under study as well as correlation analyses were performed using IBM SPSS 29. Fuzzy-set qualitative comparative analyses (fsQCA) were conducted by means of the fsQCA software (version 2.5) [43]. Finally, the PROCESS macro for SPSS (version 3.2) was used to assess the moderating effects of resilience and emotion dysregulation [47].

## Results

A total of 61 participants were included in the study. Looking at the characteristics of the sample, 45 people were female (73.80%), 15 were male (24.6%) and one person was non-binary (1.6%). Their ages ranged from 20 to 66 years (M = 43.79; SD = 12.75). Basic demographic and clinical characteristics are summarized in Table 1.

**Table 1.** **Demographic and clinical characteristics**.

Variable	Category	n	%
Marital Status	Married	26	42.60
	In a relationship	13	21.30
	Single	13	21.30
	Divorced/separated	4	6.50
	Widowed	5	8.30
Level of education	Basic education	5	8.60
	High school	13	22.40
	High school diploma or vocational training	24	41.40
	College degree	16	27.60
	Lost values	3	-
Employment Status	Unemployed	18	29.50
	Employed	25	41.00
	Students	5	8.20
	Retired	2	3.30
	Other/Not specified	11	18.00
Socioeconomic Status (annual income)	High–High (>€100,000)	0	-
	High–Medium (€45,000–€99,999)	6	10.90
	Medium–High (€25,500–€44,999)	6	10.90
	Medium–Medium (€16,000–€25,499)	15	27.30
	High–Low (€10,000–€15,999)	16	29.10
	Low–Medium (€6500–€9999)	5	9.10
	Low–Low (<€6499)	7	12.70
	Lost values	6	-
Previous Mental Health Service	Yes	36	59.30
	No	24	40.70
	Lost values	1	-
Previous Treatment Type	Combined (psychiatric & psychological)	20	55.60
	Psychological only	9	25.00
	Psychiatric only	7	19.40
Current Treatment Type	Combined (psychiatric & psychological)	31	51.20
	Psychological only	18	30.20
	Psychiatric only	11	18.60
	Lost values	1	-
Chronic Physical Illness	Yes	30	54.54
	No	25	45.45
	Lost values	6	-

### Descriptive Analysis

Firstly, regarding symptoms, the sample demonstrated elevated levels of post-traumatic stress and average levels of general psychopathology. When making the assumptions of normality, some of the variables did not follow a normal distribution (social support, post-traumatic stress and stressful life events), so Table 2 shows the data relating to the descriptive statistics. With respect to psychological variables, the participants exhibited high levels of neuroticism and agreeableness. Additionally, they demonstrated medium-to-high levels of openness to experience and conscientiousness. Finally, the participants displayed medium levels of extraversion. However, given the findings reported in the extant literature, only the dimensions of extraversion and conscientiousness were considered for the analyses. Conversely, the sample exhibited average values in emotion dysregulation, social support, resilience, and low levels of stressful life events (Table 2).

**Table 2.** **Descriptive analysis**.

	M	SD	Min	Max	Mdn	IQR	S	K
Post-traumatic stress	-	-	0	80	51.00	31.50	–0.36	–0.69
Psychopathology	1.68	0.89	0	4	-	-	0.47	–0.56
Extraversion	20.51	8.42	0	48	-	-	–0.21	–0.55
Conscientiousness	29.47	8.42	0	48	-	-	–0.34	–0.79
Emotion dysregulation	82.28	22.27	28	140	-	-	0.11	–0.49
Resilience	20.63	8.23	0	40	-	-	–0.25	–0.91
Social support	-	-	11	55	38.00	16.50	–0.22	–1.29
Stressful life events	-	-	0	9	2.00	2.00	1.35	2.33

Note. M, average; SD, standard deviation; Min, minimum; Max, maximum; Mdn, Median; IQR, interquartile range; S, Skewness; K, Kurtosis.

### Correlation Analysis

Normality was examined using the Kolmogorov–Smirnov test, along with skewness and kurtosis coefficients. Since some variables met the assumption of normality and others did not, Spearman’s correlations were used. Significant correlations were observed among the variables analyzed. Post-traumatic stress was positively correlated with psychopathology, emotion dysregulation and stressful life events, and negatively with extraversion and resilience. Psychopathology was positively associated with emotion dysregulation and negatively with extraversion, conscientiousness, resilience and social support. Resilience showed positive correlations with extraversion, conscientiousness and social support and a negative correlation with emotion dysregulation. Finally, social support was negatively correlated with emotion dysregulation and stressful life events, and positively with extraversion (Table 3).

**Table 3.** **Correlations between psychological variables**.

	1	2	3	4	5	6	7	8
Post-traumatic stress	1							
Psychopathology	0.824**	1						
Extraversion	–0.312*	–0.615***	1					
Conscientiousness	–0.263	–0.392**	0.393**	1				
Emotion dysregulation	0.653***	0.755***	–0.500***	–0.459***	1			
Social support	–0.255	–0.335*	0.304*	–0.102	–0.331**	1		
Resilience	–0.404**	–0.582***	0.576**	0.460**	–0.533**	0.269*	1	
Stressful life events	0.440**	0.370*	–0.056	–0.014	0.233	–0.248	–0.109	1

*p ≤ 0.05; **p ≤ 0.01; ***p ≤ 0.001.

### Fuzzy-Set Comparative Qualitative Analysis

First, to carry out the QCA models, the calculated calibration values are presented (Appendix Table 7). Then, necessity analyses were performed, followed by those of sufficiency, as suggested in the literature. Post-traumatic stress and psychopathology were established as the criterion variable (outcome condition according to QCA terminology).

### Necessary Analysis

Based on the results obtained in the necessary analysis, there was no necessary condition for the high or low levels of post-traumatic stress based on the studied variables, since all consistency values were below 0.90 [43]. Regarding psychopathology, low levels of extraversion were found as the only necessary condition of psychopathology (consistency = 0.09; coverage = 0.62).

### Sufficiency Analysis

Regarding the sufficiency analysis, resulting models for post-traumatic stress and psychopathology were shown in Table 4 (Ref. [48]), based on the premise that in QCA a model is informative when the consistency is above 0.74 [43].

**Table 4.** **Sufficiency analysis for post-traumatic stress and psychopathology**.

Frequency cut-off 1	High			Low			High			Low		
	Post-traumatic stress			Post-traumatic stress			Psycho-pathology			Psycho-pathology		
	Consistency Cut-off: 0.80			Consistency Cut-off: 0.82			Consistency Cut-off: 0.81			Consistency Cut-off: 0.93		
	1	2	3	1	2	3	1	2	3	1	2	3
Extraversion	○	○	○			●	○	○	○		●	
Conscientiousness		○	○					○	○			
Emotion dysregulation	●	●	●	○	○		●	●		○		○
Resilience		○			○	○		○		●		
Social support	○			●	●		○					●
Stressful life events			●	○		○			●	○	○	○
Raw coverage	0.60	0.55	0.45	0.44	0.38	0.32	0.65	0.62	0.58	0.50	0.49	0.46
Unique coverage	0.05	0.05	0.02	0.06	0.05	0.09	0.04	0.04	0.09	0.09	0.05	0.05
Consistency	0.87	0.86	0.95	0.85	0.86	0.90	0.87	0.87	0.84	0.94	0.96	0.91
Total consistency			0.74			0.83			0.77			0.91
Total coverage			0.78			0.67			0.88			0.73

Note. ○, absence of condition; ●, presence of condition Expected vector for high post-traumatic stress and psychopathology: 1.0.0.0.1.0. Expected vector for low post-traumatic stress and psychopathology: 0.1.1.1.0.1 [48].

Regarding post-traumatic stress models, for high levels of post-traumatic stress, seven pathways explained 78% of cases (overall consistency = 0.74; overall coverage = 0.78). The three most relevant pathways were: (a) low social support, low extraversion and high emotion dysregulation (consistency = 0.87; coverage = 0.60); (b) low resilience, low extraversion, low conscientiousness and high emotion dysregulation (consistency = 0.86; coverage = 0.55); and (c) high stressful life events, low extraversion, low conscientiousness and high emotion dysregulation (consistency = 0.95; coverage = 0.45). These combinations explained 60%, 55% and 45% of high post-traumatic stress cases, respectively.

For low levels of post-traumatic stress, five pathways explained 67% of cases (overall consistency = 0.83; overall coverage = 0.67). The three most relevant were: (a) high social support, low stressful life events and low emotion dysregulation (consistency = 0.85; coverage = 0.44); (b) high social support, low resilience and low emotion dysregulation (consistency = 0.86; coverage = 0.38); and (c) low stressful life events, low resilience and high extraversion (consistency = 0.90; coverage = 0.32). These explained 44%, 38% and 32% of the low post-traumatic stress cases, respectively.

### Moderation Models 

The moderating role of emotion dysregulation and resilience was evaluated.

#### Emotion Dysregulation as a Moderate Variable

First, an analysis was conducted to test whether emotion dysregulation acts as a modulator in the relationship of stressful life events and psychopathology and post-traumatic stress symptomatology. Regarding post-traumatic stress, emotion dysregulation did not function as a modulator in the relationship between stressful life events and post-traumatic stress symptomatology.

**Fig. 2.**
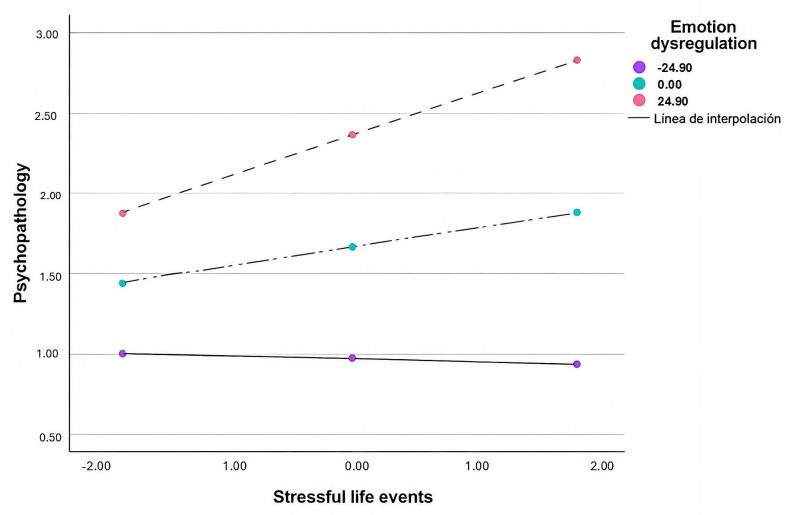
**Moderating effects of emotion dysregulation on the relationship between stressful life events and psychopathology**. Note. Stressful life events = independent variable; Emotion dysregulation = moderating variable. Psychopathology = dependent variable. Low Emotion dysregulation (Mean – 1SD); Medium Emotion dysregulation (Mean); High Emotion dysregulation (Mean + 1SD).

Regarding psychopathology (Table 5), the results indicated that the moderating model explained the 64% of psychopathology variance. The inclusion of the interaction term stressful life events × emotion dysregulation produced a significant increase in the explained variance of the model, ΔR^2^ = 0.048, F (1, 36) = 4.83, *p* = 0.04. This result indicates that emotion dysregulation moderated the relationship between stressful life events and psychopathology. With regards to the conditional effects, the relationship of stressful life events and psychopathology was significant for medium (t = 2.38; *p* = 0.02; 95% CI = [0.02,0.23]) and high (t = 3.04; *p* = 0.004; 95% CI = [0.09,0.44]) levels of emotion dysregulation (Fig. 2) but it was not significant for low levels of deregulation.

**Table 5.** **Moderating effects of emotion dysregulation on the relationship between stressful life events and psychopathology**.

		Effect	SE	*t*	*p*	LLCI	ULCI
Model for psychopathology							
R^2^ = 0.64; F (3,36) = 21.48; *p * ≤ 0.001							
	Stressful life events	0.12	0.05	2.38	0.02	0.02	0.23
	Emotion dysregulation	0.03	0.003	7.44	0.001	0.02	0.04
	Stressful life events X Emotion dysregulation	0.01	0.002	2.20	0.03	0.004	0.01
Conditional effects							
	Low (DERS = –24.90)	–0.02	0.08	–0.29	0.78	–0.18	0.14
	Medium (DERS = 0.00)	0.12	0.05	2.38	0.02	0.02	0.23
	High (DERS = 24.90)	0.27	0.09	3.04	0.004	0.09	0.44

Note. SE, Standard Error; LLCI, Lower Level Confidence Interval; ULCI, Upper Level Confidence Interval.

#### Resilience as a Moderate Variable

Second, an analysis was conducted to test whether resilience acts as a modulator in the relationship of emotion dysregulation and psychopathology and post-traumatic stress symptomatology.

**Fig. 3.**
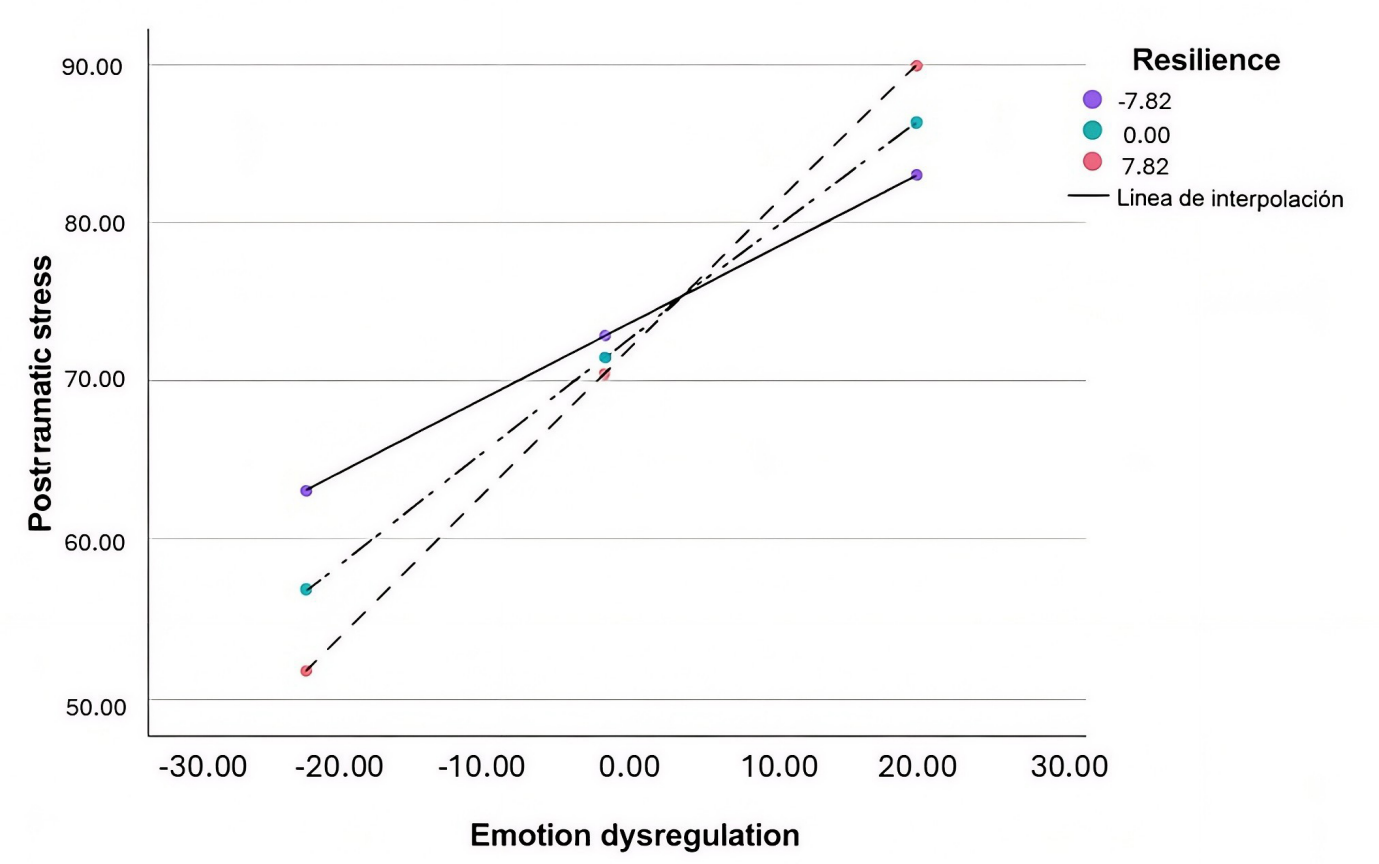
**Moderating effects of resilience on the relationship between emotion dysregulation and post-traumatic stress**. Note. Emotion dysregulation = independent variable; Resilience = moderating variable. Post-traumatic stress = dependent variable. Low Resilience (Mean – 1SD); Medium Resilience (Mean); High Resilience (Mean + 1SD).

Regarding post-traumatic stress (Table 6), resilience functioned as a moderator in the relationship between emotion dysregulation and post-traumatic stress symptomatology, even though its main effect in the initial model was not significant. The moderation model explained 52% of the variance in post-traumatic stress. The inclusion of the interaction term emotion dysregulation × resilience produced a significant increase in the explained variance of the model, ΔR^2^ = 0.094, F (1, 43) = 4.42, *p* = 0.04. This result indicates that emotion dysregulation moderated the relationship between stressful life events and psychopathology. Conditional effects analyses showed that the relationship between emotion dysregulation and post-traumatic stress was significant at all levels of resilience: low (t = 3.27; *p* = 0.002; 95% CI = [0.17, 0.73]), medium (t = 5.44; *p* < 0.001; 95% CI = [0.41, 0.90]), and high (t = 5.05; *p* < 0.001; 95% CI = [0.52, 1.20]) (Fig. 3). As resilience increases, the impact of emotion dysregulation on post-traumatic stress also increases, indicating that the relationship becomes stronger or more pronounced. These results confirm that resilience moderates the effect of emotion dysregulation on post-traumatic stress symptomatology, even though it does not have a significant direct effect on stress symptoms.

**Table 6.** **Moderating effects of resilience on the relationship between emotion dysregulation and post-traumatic stress**.

		Effect	SE	*t*	*p*	LLCI	ULCI
Model for post-traumatic stress							
R^2^ = 0.52; F (3,43) = 15.48; *p * ≤ 0.001							
	Emotion dysregulation	0.65	0.12	5.44	0.001	0.41	0.90
	Resilience	–0.16	0.33	–0.48	0.63	–0.82	0.51
	Emotion dysregulation X Resilience	0.03	0.01	2.10	0.04	0.001	0.05
Conditional effects							
	Low (Resilience = –7.82)	0.45	0.14	3.27	0.002	0.17	0.73
	Medium (Resilience = 0)	0.65	0.12	5.44	0.001	0.41	0.90
	High (Resilience = 7.82)	0.86	0.17	5.05	0.001	0.52	1.20

Note. SE, Standard Error; LLCI, Lower Level Confidence Interval; ULCI, Upper Level Confidence Interval.

In contrast, regarding general psychopathology, resilience did not function as a moderator in the relationship between emotion dysregulation and psychopathology.

## Discussion

The aim of this study was to analyze how transdiagnostic factors (emotion dysregulation, personality traits, resilience, stressful life events, and social support) are associated with post-traumatic stress and psychopathology in a clinical adult population in Spain, using correlational, qualitative QCA and PROCESS models.

Mental disorders affect a significant proportion of the population and often co-occur, challenging categorical diagnostic models and supporting the development of dimensional approaches such as the transdiagnostic model, which emphasize shared underlying processes [7, 8, 11]. As expected, the clinical sample showed elevated levels of significant psychological symptomatology [20, 21, 22, 24]. Specifically, high levels of post-traumatic stress symptoms and psychopathology were observed, alongside average levels of emotion dysregulation, resilience, conscientiousness, and social support, and low levels of extraversion. Surprisingly, the sample did not report high levels of stressful life events.

Our first hypothesis proposed that symptoms of post-traumatic stress and psychopathology would be associated with stressful life events, emotion dysregulation, low resilience, low extraversion, low conscientiousness, and low social support. Based on our correlational analyses, consistent with prior research [16, 17, 18], emotion dysregulation emerged as a central transdiagnostic factor: it was positively correlated with both psychopathology and post-traumatic stress, and negatively correlated with resilience, social support, extraversion, and conscientiousness. Similarly, in line with previous findings [20, 21], resilience and extraversion were negatively associated with post-traumatic stress and psychopathology. Therefore, the results from linear analyses support the presence of transdiagnostic factors that contribute to the development of psychopathological symptoms.

Considering the QCA results, no necessary conditions were identified to explain high or low levels of post-traumatic stress symptomatology. However, in the case of psychopathology, one necessary condition did emerge: low levels of extraversion, which were associated with higher symptomatology. This finding may be understood in light of evidence suggesting that low extraversion functions as a vulnerability factor by depriving individuals of key emotion and social resources for coping with adverse situations [14], thereby increasing the likelihood of developing or maintaining psychopathological symptoms. In addition, the sufficiency analyses revealed several combinations of factors that accounted for the variability observed in both post-traumatic stress symptomatology and psychopathology. Across these configurations, emotion dysregulation consistently appeared as a core component of high-risk profiles [17, 18]. Specifically, profiles characterized by higher levels of post-traumatic stress and psychopathology tended to combine low social support, low extraversion, and high emotion dysregulation. Conversely, profiles with lower levels of post-traumatic stress were associated with lower exposure to stressful life events, high social support, and low emotion dysregulation, while profiles with lower levels of psychopathology were linked to lower exposure to stressful life events, high resilience, and low emotion dysregulation.

The combination of these different methodologies proves highly valuable for identifying both general patterns and specific differences. On the one hand, linear analyses such as correlations help reveal how variables are associated with one another. On the other hand, approaches like the QCA model make it possible to explore which combinations of variables may converge on the same outcome, accounting for multiple alternative pathways. Taken together, emotion dysregulation emerged as a central element across most explanatory configurations. These analyses offer a flexible and enriched understanding of transdiagnostic processes, which is highly relevant both for advancing research and informing clinical interventions.

Following hypothesis two, it was proposed that the impact of stressful life events on post-traumatic stress and psychopathology would be moderated by emotion regulation. Our results partially supported this hypothesis. Previous studies have shown that emotion regulation can moderate the relationship between stressful life events and both stress responses and psychopathological outcomes [27, 28]. In our study, however, emotion dysregulation only showed a moderating effect on the relationship between stressful life events and psychopathology, but not on post-traumatic stress symptomatology. Specifically, individuals who had experienced a greater number of stressful life events reported significantly higher levels of psychopathological symptoms when they also presented higher emotion dysregulation. These findings further reinforce the transdiagnostic role of emotion regulation [30, 31], underlining its importance as a key target for both prevention and psychological intervention strategies aimed at reducing vulnerability to psychopathology. It is imperative to acknowledge the potential influence of gender and cultural factors on emotion regulation processes, which could impact the interpretation of the present findings. The sample in this study was predominantly female (73.8%) and regionally restricted, a limitation that potentially restricts the generalizability of the results. According to extant research, women tend to report a greater degree of emotion awareness and intensity, as well as a higher use of certain emotion regulation strategies, such as rumination or expressive suppression, compared to men [49]. These disparities may be shaped not only by biological predispositions but also by sociocultural norms governing emotion expression and coping. A number of studies have been conducted in Spain to examine emotion regulation and resilience in both clinical and general adult populations. For instance, research on Spanish adults has found higher levels of resilience and adaptive regulation strategies compared to other populations [50, 51]. However, these studies primarily focus on non-clinical or community samples and rarely examine the interaction between emotion regulation, resilience, and psychopathology [50]. To date, there is a notable absence of cross-cultural studies involving Spanish clinical populations that investigate how these mechanisms operate in comparison to those observed in other countries. Consequently, the moderating role of emotion regulation may differ across genders and cultures. Future studies should use more gender-balanced and culturally diverse clinical samples, including cross-national comparisons, to clarify how emotion regulation and resilience influence psychopathology. Finally, according to our third hypothesis, it was proposed that the effect of emotion regulation on both post-traumatic stress and psychopathology would be moderated by resilience. Interestingly, although resilience has been widely described in the literature as a protective moderator against the negative impact of stress [23], our results showed a different pattern. On the one hand, resilience did not play a moderating role in the relationship between emotion dysregulation and psychopathology. On the other hand, resilience moderated the relationship between emotion dysregulation and post-traumatic stress symptomatology. Contrary to what might be expected, the effect of emotion dysregulation on post-traumatic stress symptoms was stronger at higher levels of resilience. In other words, instead of buffering the adverse impact of emotion dysregulation, resilience seemed to amplify it: individuals with high emotion dysregulation reported more severe post-traumatic stress symptoms when their resilience was also high. This counterintuitive finding might suggest that when emotion regulation capacities are severely impaired, even relatively high resilience is insufficient to counterbalance this vulnerability. It is possible that greater resilience, in this context, increases awareness of distress or creates a mismatch between the perceived capacity to cope and actual difficulties in regulating emotions, ultimately exacerbating symptom severity. Contrary to prevailing assumptions, the impact of emotion dysregulation on post-traumatic stress symptoms manifested with greater intensity among individuals who demonstrated higher levels of resilience. In essence, the study found that, rather than mitigating the adverse effects of emotion dysregulation, resilience appeared to amplify them. Individuals with high levels of emotion dysregulation exhibited more pronounced post-traumatic stress symptoms when their resilience levels were also high.

This counterintuitive finding suggests that when emotion regulation capacities are severely impaired, even relatively high resilience may be insufficient to counterbalance this vulnerability. One possible interpretation of these findings draws on the concept of “resilience fatigue” [52, 53], which proposes that the sustained effort to remain resilient in the face of chronic stress may deplete psychological resources over time, ultimately increasing vulnerability to psychopathology. Another potential explanation involves a discrepancy between perceived and actual coping capacities [29]. Individuals who perceive themselves as resilient may underestimate their emotion difficulties, leading to internal conflict or delayed help-seeking when facing trauma-related distress. In such cases, resilience may function more as a perceived trait than an effective regulatory capacity, which could exacerbate symptom severity when emotion dysregulation is high. Future research should explore the dynamic interplay between resilience and emotion regulation, distinguishing between adaptive and potentially maladaptive aspects of resilience under extreme stress conditions.

Despite the valuable contributions of this study, several limitations should be acknowledged. First, the correlational cross-sectional design restricts causal inferences; future research would benefit from longitudinal or clinical studies to provide a more comprehensive understanding. Second, the non-probabilistic sampling method, the predominance of female participants, and the geographic restriction to the Valencian Community limit the generalizability of the findings. Third, although the questionnaires used demonstrated adequate psychometric properties and counterbalancing procedures were implemented, the potential influence of social desirability bias cannot be ruled out. Additionally, the relatively modest sample size (n = 61) may have reduced statistical power to detect moderate associations, affecting the precision and generalizability of the results. These limitations highlight the need for cautious interpretation and replication of findings in larger, more diverse, and prospectively designed samples.

## Conclusions

The findings of this study contribute to the transdiagnostic perspective by suggesting that factors such as emotion regulation may play a significant role not only in the manifestation of clinical symptomatology but also as potential moderators in the association between risk variables and mental health outcomes. This underscores the relevance of examining shared psychological processes that transcend traditional diagnostic boundaries, which may otherwise constrain our understanding of the multifaceted nature of mental disorders.

In conclusion, these results support the importance of developing interventions that strengthen emotion regulation skills and other protective factors, as part of more integrative prevention and clinical frameworks aimed at common underlying mechanisms across psychopathological conditions. However, given the cross-sectional nature of the present study, causal inferences should be made with caution. Future longitudinal and experimental research would be valuable to clarify the temporal and causal dynamics between these transdiagnostic factors and mental health outcomes.

## Availability of Data and Materials

The data are available from the corresponding author, [SVM], upon reasonable request.

## Appendix

See Table 7.

**Table 7.** **Main descriptor and calibration values**.

	M	SD	P10	P50	P90	Min	Max
Post-traumatic stress	70.49	20.33	38	73	96	22	99
Psycho-pathology	14.61	11.52	5.31	13.48	25.60	0.96	0.86
Extraversion	3,871,501.09	16,687,891.64	234	77,280	4,860,000	6	11,652,336
Conscien-tiousness	70,730,675,225	5.28147 × 10^11^	300	3.99 × 10^22^	8150.40	4,320,000	261,601,344.80
Emotion dysregulation	123,577.39	340,402.99	184.50	9092	300,622.50	12	2,032,525
Resilience	309,712.75	619,091.32	64	34,560	988,932	4	3,750,000
Social support	1,730,776.36	3,608,903.35	556	221,778	6,000,000	48	17,578,125
Stressful life events	21.62	74.25	1	4	32	1	512

Note. M, average; SD, standard deviation; P10, percentile 10; P50, percentile 50; P90, percentile 90; Min, minimum; Max, maximum.
